# Use of complementary and alternative medicine in patients with inborn errors of metabolism: A single‐center study

**DOI:** 10.1002/jmd2.12089

**Published:** 2019-12-18

**Authors:** Jessica Tao, C. Anthony Rupar, Michael R. Miller, Suzanne Ratko, Chitra Prasad

**Affiliations:** ^1^ Faculty of Science Western University London Ontario Canada; ^2^ Department of Pathology & Laboratory Medicine and Biochemistry Western University London Ontario Canada; ^3^ Department of Paediatrics Western University London Ontario Canada; ^4^ Children's Health Research Institute, Western University London Ontario Canada; ^5^ Children's Hospital, London Health Sciences Centre London Ontario Canada

**Keywords:** complementary and alternative medicine, complementary therapies, inborn errors of metabolism, integrative medicine, surveys and questionnaires

## Abstract

**Background and Objectives:**

There is a paucity of information on the use of complementary and alternative medicine (CAM) in patients with inborn errors of metabolism (IEM). This study's objective was to evaluate the self‐reported use and perceived effectiveness of CAM in adults and children with IEM.

**Methods:**

Patients aged 0‐70 years and caregivers seen at the London Health Sciences Centre Metabolic Clinic (London, Ontario, Canada) between July 2017 and August 2017 were recruited to complete a questionnaire regarding CAM use to help their IEM diagnosis and perceived effectiveness of these therapies. Survey responses were analyzed using descriptive statistics; age, sex, and education level associations among CAM users were tested using the Pearson *χ* ^2^ test.

**Results:**

Of 50 potential participants, 44 (88%) completed the questionnaire, including 21 adults (6 by caregivers) and 23 children (22 by caregivers). The most common IEM category was Aminoacidopathies and Small Molecule Disorders (50%). Twenty‐seven (61%) participants reported CAM use to help their IEM diagnosis. The most common CAM therapies used were chiropractic manipulation, omega‐3 fatty acids, probiotics, and aromatherapy/essential oils. Most CAM users and caregivers (74%) perceived their CAM therapies as effective overall. Among CAM users, 40% had not discussed CAM use with a health care professional (HCP). CAM use was similar when comparing age, sex and education level.

**Conclusions:**

CAM is commonly used among patients with IEM. The safety and efficacy of CAM therapies for IEM should be further investigated. HCPs and patients should openly discuss CAM use in order to evaluate safety.

## 1 INTRODUCTION

Complementary and alternative medicine (CAM) is defined by the National Center for Complementary and Integrative Health (NCCIH) as “a group of diverse medical and health care systems, practices, and products that are not generally considered part of conventional medicine”.^1^ CAM therapies include, but are not limited to, natural products (eg, herbal supplements, probiotics), mind and body practices (eg, chiropractic manipulation, yoga, meditation), and other health approaches (eg, Traditional Chinese Medicine, naturopathy).^2^ There is a high prevalence of CAM use in North America with 79% of Canadians in 2016 reporting having tried CAM in their lifetime.^3^, ^4^ Additionally, many caregivers find CAM beneficial and prefer it over conventional medicine for their children.^5^ However, it is often overlooked that CAM may lead to adverse events.^6^, ^7^, ^8^

There remains a paucity of information on the use and effectiveness of CAM in patients with inborn errors of metabolism (IEM), a group of over 500 disorders that affects approximately 50.9 in 100 000 live births globally.^9^ Since IEM are individually rare, conventional treatments are limited for many disorders; therefore, many patients with IEM may use CAM to treat symptoms and improve their quality of life. Recent CAM use estimates include 41% of patients with IEM in Turkey,^10^ and nearly half of patients with lysosomal storage diseases in the United States.^11^ Additionally, recent studies have investigated the effects of individual CAM therapies on specific IEM disorders, such as acupuncture in patients with Gaucher disease.^12^ However, less than 15% of CAM users with IEM have reported discussing their CAM use with their physician.^10^ Many IEM disorders present with significant systemic manifestations and are treated with physician‐prescribed dietary therapy. Use of certain CAM therapies in patients with IEM may be of particular concern as some CAM therapies have been associated with life‐threatening adverse effects such as organ toxicities and mechanical injuries.^13^, ^14^, ^15^ Therefore, it is important for physicians and allied health professionals to be aware of the prevalence of CAM use, and openly discuss CAM use with their patients with IEM in order to evaluate safety and possible adverse interactions.

This study aimed to investigate the self‐reported use and perceived effectiveness of CAM in adults and children with IEM, and the factors associated with using these therapies. This study also provides preliminary information of CAM use and perceived effectiveness in patients with IEM in Canada.

## 2 METHODS

A cross‐sectional questionnaire study was conducted on adult and pediatric patients with IEM aged 0 to 70 years who attended their London Health Sciences Centre (LHSC) Metabolic Clinic appointment in London, Ontario, Canada, between July 2017 and August 2017. The questionnaire (Supplementary Material S1) was developed de novo after reviewing similar surveys^11^, ^16^, ^17^, ^18^ and included questions regarding the patient's age, sex, ethnicity, education level, IEM diagnosis, and experiences with CAM. Caregivers present at the appointment completed the questionnaire for patients who were unable to independently do so, and their education level was also obtained. IEM diagnoses were organized into categories for patient data privacy and statistical purposes.

Participants were asked about past and present use of 15 CAM supplements and 21 CAM treatments/practices to help their IEM diagnosis, which were selected based on previous relevant studies and clinical experience (Supplementary Material S1). Participants also had the option to include unlisted CAM therapies that they have used. A participant was classified as a “CAM User” if they reported use of at least one CAM therapy to help their IEM diagnosis. CAM Users were then asked to report their perceived effectiveness for each used CAM therapy using a Likert scale of 0‐5, with 0 being “not effective at all”, and 5 being “very effective.” A CAM therapy was classified as “perceived effective” if it received a median score of 3 or above on the Likert scale. If a CAM User received a mean score of 3 or above for all their used CAM therapies, their individual CAM use was considered as “perceived effective” overall. The questionnaire also included questions about whether CAM Users had discussed their CAM use with a health care professional (HCP) and associated financial costs.

The questionnaire data were collected using Microsoft Excel and analyzed using SPSS version 24 (IBM Corp., Armonk, New York). Descriptive statistics were obtained to examine demographic and disease characteristics, as well as prevalence and perceptions of CAM use. Pearson *χ* ^2^ tests were used to determine age, sex and education level associations among CAM Users; *P* < .05 was considered statistically significant. This study was approved by the Western University Research Ethics Board and Lawson Health Research Institute (London, Ontario, Canada).

## 3 RESULTS

### 3.1 Study population

During the study period, 50 patients were approached to complete the questionnaire, and 44 (88%) agreed to participate. The demographic and diagnosis characteristics of the study population are described in Table 1. A total of 52% of participants were children aged 0‐17 years and 52% of participants were male. The questionnaire was completed by caregivers for 29% and 96% of our adult and pediatric participants respectively. Twenty‐four IEM disorders were represented in the study's cohort. The most common reported IEM diagnosis category was Aminoacidopathies and Small Molecule disorders (50%), and Phenylketonuria was the most common diagnosis (20%). The majority of participants were Caucasian (91%), and most individuals who completed the questionnaire (ie, patients who independently completed the questionnaire and caregivers who completed the questionnaire on the patient's behalf) had some postsecondary education or higher (66%).

**Table 1:** Demographic characteristics, diagnosis distribution and CAM use of participants

	All participants (N = 44)	Children (0‐17 years) (N = 23)	Adults (18‐70 years) (N = 21)
Questionnaire completed by caregiver, n (%)	28 (64%)	22 (96%)	6 (29%)
Sex, n (%)			
Male	23 (52%)	13 (57%)	10 (48%)
Female	21 (48%)	10 (43%)	11 (52%)
IEM diagnosis, n (%)			
Aminoacidopathies and Small Molecule disorders^a^	22 (50%)	15 (65%)	7 (33%)
Energy disorders and Mitochondrial disorders^b^	8 (18%)	4 (17%)	4 (19%)
Lysosomal storage disorders^c^	5 (11%)	2 (9%)	3 (14%)
Carbohydrate disorders^d^	3 (7%)	1 (4%)	2 (10%)
Miscellaneous^e^	6 (14%)	1 (4%)	5 (24%)
Ethnicity, n (%)			
Caucasian	40 (91%)	20 (87%)	20 (95%)
South East Asian	1 (2%)	1 (4%)	0 (0%)
Aboriginal	1 (2%)	1 (4%)	0 (0%)
Other^f^	2 (5%)	1 (4%)	1 (5%)
Education level of questionnaire completer, n (%)			
High school diploma or less	15 (34%)	8 (35%)	7 (33%)
Some postsecondary education or higher	29 (66%)	15 (65%)	14 (67%)
Use of CAM, n (%)			
Yes	27 (61%)	13 (57%)	14 (67%)
No	17 (39%)	10 (43%)	8 (38%)

*Note*: No participants reported the following ethnicities: Arab, Black, Chinese, Latin American, South Asian.

Abbreviations: CAM, complementary and alternative medicine; IEM, inborn errors of metabolism.

a Aminoacidopathies and Small Molecule disorders include Arginase deficiency, Argininosuccinate lyase (ASL) deficiency, Biotinidase deficiency, Glucose transporter 1 (GLUT1) deficiency, Methylenetetrahydrofolate reductase (MTHFR) deficiency, Methylmalonic acidemia, Ornithine transcarbamylase (OTC) deficiency (including carriers), Phenylketonuria (PKU), and Pyridoxine‐dependent epilepsy (PDE).

b Energy disorders and Mitochondrial disorders include Medium‐chain acyl‐CoA dehydrogenase deficiency (MCADD), Mitochondrial encephalomyopathy, lactic acidosis, and stroke‐like episodes (MELAS), Mitochondrial disease, and Mitochondrial myopathy.

c Lysosomal storage disorders include Fabry, Gaucher, Lysosomal acid lipase deficiency (LAL‐D), Mucopolysaccharidosis type IVA (MPS IVA), and Pompe.

d Carbohydrate disorders include Classic galactosemia, and Glycogen storage disease type 1A.

e Miscellaneous disorders include Acute intermittent porphyria (AIP), Congenital disorder of glycosylation type 1A (CDG1A), Growth hormone deficiency and Developmental delay, and Smith‐Lemli‐Opitz syndrome.

f Other ethnicities reported include multi‐ethnic, or participant did not disclose.

### 3.2 Use of CAM

Twenty‐seven (61%) of the 44 participants reported CAM use to help their IEM diagnosis. Of the 27 CAM Users, 13 (48%) were children and 14 (52%) were adults (Table 1). Table 2 describes the characteristics of CAM use by our CAM Users. The most frequently reported reasons for using CAM included “To relieve symptoms” (76%, N = 17), “To complement my prescribed medical therapy” (41%, N = 17), and “To try something new/different” (35%, N = 17) (Table 2). An average of four different CAM therapies was used among CAM Users. The most commonly used CAM supplements were omega‐3 fatty acids (37%), probiotics (37%), and megavitamin therapy (22%), and the most commonly used CAM treatments/practices were chiropractic manipulation (41%) and aromatherapy/essential oils (30%) (Table 3). Eight (40%, N = 20) CAM Users claimed that they had not discussed their CAM use with a conventional HCP such as physicians, dieticians, nurses, or pharmacists; the majority of these respondents were adults (63%, N = 8) (Table 2). CAM Users most frequently reported spending $0‐$100 CAD per month on CAM (75%, N = 16) (Table 2). Among CAM Users, there were no significant differences between CAM use and age (children vs adults, *P* = .490), sex (males vs females, *P* = .490), or education level of the questionnaire completer (high school diploma or less vs some postsecondary education or higher, *P* = .603).

**Table 2:** Characteristics of CAM use

	All CAM Users (N = 27)	Children (0‐17 years) (N = 13)	Adults (18‐70 years) (N = 14)
Reasons for using CAM, n (%)	(N = 17)	(N = 9)	(N = 8)
To relieve symptoms	13 (76%)	6 (67%)	6 (75%)
To complement my prescribed medical therapy	7 (41%)	4 (44%)	3 (38%)
To try something new/different	6 (35%)	3 (33%)	3 (38%)
My CAM therapies make me feel better	4 (24%)	1 (11%)	3 (38%)
Not satisfied with my prescribed medical therapy	4 (24%)	3 (33%)	1 (13%)
Suggested by a conventional medical professional	3 (18%)	0 (0%)	3 (38%)
Discussed CAM use with a HCP, n (%)	(N = 20)	(N = 11)	(N = 9)
Yes	12 (60%)	8 (73%)	4 (44%)
No	8 (40%)	3 (27%)	5 (56%)
HCPs that participants have discussed CAM use with, n (%)	(N = 12)	(N = 8)	(N = 4)
Physician	8 (67%)	4 (50%)	4 (100%)
Dietician	5 (42%)	5 (63%)	0 (0%)
Physiotherapist	4 (33%)	2 (25%)	2 (50%)
Cost of CAM per month, n (%)	(N = 16)	(N = 9)	(N = 7)
$0‐100 CAD	12 (75%)	6 (67%)	6 (86%)
$100‐400 CAD	4 (25%)	3 (33%)	1 (14%)

*Notes*: No participants reported using CAM “to combat the prescribed medical therapy's side effect(s),” “because prescribed medical therapy is too expensive.” No participants reported discussing CAM use with a genetic counselor, nurse practitioner, pharmacist, registered nurse, other HCP.

Abbreviations: CAD, Canadian dollar; CAM, complementary and alternative medicine; HCP, health care professional.

**Table 3:** Most common CAM supplements and treatments/practices used by patients with IEM

CAM therapy	All CAM Users, n (%) (N = 27)	Median perceived effectiveness^a^ (IQR)	Children (0‐17 years), n (%) (N = 13)	Adults (18‐70 years), n (%) (N = 14)
Supplements				
Antioxidants	3 (11%)	2 (1.5‐3.5)	2 (15%)	1 (7%)
Echinacea	4 (15%)	3 (2.25‐3)	0 (0%)	4 (29%)
Fenugreek	1 (4%)	3 (3‐3)	0 (0%)	1 (7%)
Flaxseed	5 (19%)	3 (3‐4)	3 (23%)	2 (14%)
Garlic supplements	4 (15%)	4 (4‐4.25)	3 (23%)	1 (7%)
Ginseng	2 (7%)	2 (1‐3)	0 (0%)	2 (14%)
Glucosamine (only)	2 (7%)	0 (0‐0, n = 1)	1 (8%)	1 (7%)
Megavitamin therapy	6 (22%)	4 (3.25‐4)	3 (23%)	3 (21%)
Omega‐3 fatty acids	10 (37%)	3 (2‐3.75)	7 (54%)	3 (21%)
Prebiotics	1 (4%)	3 (3‐3)	1 (8%)	0 (0%)
Probiotics	10 (37%)	3.5 (3‐5)	5 (38%)	5 (36%)
Other^b^	4 (15%)	2 (2‐2, n = 5)	3 (23%)	1 (7%)
Treatments/practices				
Acupressure	1 (4%)	4 (4‐4)	0 (0%)	1 (7%)
Acupuncture	2 (7%)	3.5 (3.25‐3.75)	0 (0%)	2 (14%)
Aromatherapy/essential oils	8 (30%)	4 (4‐4)	3 (23%)	5 (36%)
Chiropractic	11 (41%)	4 (3.5‐5)	4 (31%)	7 (50%)
Energy healing/Reiki	2 (7%)	3 (3‐3)	1 (8%)	1 (7%)
Guided imagery	2 (7%)	4 (3.5‐4.5)	0 (0%)	2 (14%)
Massage therapy	4 (15%)	5 (4.75‐5)	1 (8%)	3 (21%)
Meditation	4 (15%)	4.5 (3.5‐5)	0 (0%)	4 (29%)
Naturopathy	2 (7%)	3 (2.5‐3.5)	2 (15%)	0 (0%)
Reflexology	1 (4%)	3 (3‐3)	1 (8%)	0 (0%)
Spiritual healing by others	4 (15%)	4 (3.25‐4.25)	0 (0%)	4 (29%)
Water therapy	4 (15%)	4 (3.75‐4.25)	2 (15%)	2 (14%)
Yoga	3 (11%)	3 (3‐3.5)	0 (0%)	3 (21%)
Other^c^	5 (19%)	5 (4‐5, n = 7)	2 (15%)	3 (21%)

*Notes*: No participants reported use of genistein, ginkgo, glucosamine + chondroitin, MSM. No participants reported use of Ayurveda, biofeedback, cupping therapy, homeopathy, magnetic therapy, osteopathy, progressive relaxation, and Traditional Chinese Medicine.

Abbreviations: CAM, complementary and alternative medicine; IEM, inborn errors of metabolism; IQR, interquartile range (first–third quartile); MCT, medium‐chain triglyceride; MSM, methylsulfonylmethane.

a Likert scale from 0 to 5.

b Other CAM supplements used by participants include cannabis/hemp oil, digestive enzymes, dragon fruit, ginger, MCT oil. Participants were allowed to report use of more than one “Other” CAM supplement.

c Other CAM treatments/practices used by participants include music therapy, physical activity, REID diet, and self‐help groups. Participants were allowed to report use of more than one “Other” CAM treatment/practice.

### 3.3 Perceived effectiveness of CAM therapies

Most CAM Users and caregivers (74%) perceived their own CAM therapies as effective in helping with their IEM diagnosis and associated symptoms. Of the 15 CAM supplements listed in the questionnaire, 8 received a median Likert scale score of 3 or above, and were therefore considered as “perceived effective”: garlic supplements, megavitamin therapy, probiotics, echinacea, fenugreek, flaxseed, omega‐3 fatty acids, and prebiotics (Table 3). Antioxidants, ginseng, and glucosamine were not perceived as effective by CAM Users (Table 3). Additionally, all 14 CAM treatments/practices listed in the questionnaire that CAM Users reported using were considered as “perceived effective”: massage therapy, meditation, acupressure, aromatherapy/essential oils, chiropractic manipulation, guided imagery, spiritual healing by others, water therapy, acupuncture, energy healing/reiki, naturopathy, reflexology, and yoga (Table 3).

## 4 DISCUSSION

This study describes CAM use in adults and children with IEM aged 0‐70 years from a Canadian metabolic center, perceived effectiveness of over 20 CAM therapies, and factors associated with using these therapies. Following a high response rate (88%), our study found a 61% prevalence of CAM use in patients with IEM. This was a higher prevalence than in studies by Erdol and Saglam (41.1%) and Balwani et al (40%, 42%, and 41% for patients with Type 1 Gaucher disease, Fabry disease, and Type B Niemann‐Pick disease, respectively). This was also higher than overall CAM use in the United States (33.2% of adults, 11.6% of children)^17^, ^18^ and Europe (25.9%),^19^ but lower than Canadian estimates (79% of adults, 62% of children).^3^, ^20^ This variability in CAM use prevalence may be due to differences in acceptability, accessibility, cost, and insurance coverage of CAM in different countries, as well as inconsistencies in the classification of CAM. Nonetheless, our study provides further evidence supporting the high use of CAM in patients with IEM.

Participants most commonly used CAM to relieve symptoms and complement their prescribed medical therapy. This is consistent with similar studies, as most patients use CAM together with their prescribed conventional therapy and not in place of, which is also known as integrative medicine.^2^, ^21^, ^22^ However, about one‐fourth of participants reported using CAM because they were not satisfied with their conventional medical therapy, or lack thereof. Studies have shown that patients may use CAM because they gradually lose confidence or satisfaction in conventional care for chronic diseases.^23^, ^24^ CAM use was also higher in patients with an untreatable IEM disorder,^10^ which includes disorders with limited conventional therapy options. Despite the use of CAM, it is important that IEM patients do not stop their conventional therapy when it is available, especially for IEM disorders that can lead to toxic metabolites (eg, PKU, MCADD, Galactosemia) or energy deficiencies (eg, MCADD, Mitochondrial disorders).

The most commonly used CAM therapies were chiropractic manipulation, omega‐3 fatty acids, probiotics, aromatherapy/essential oils, megavitamin therapy, and flaxseed. This is consistent with fish oil (rich in omega‐3 fatty acids) being the most commonly used natural product in the United States,^17^, ^18^ and chiropractic manipulation being popular in Canada and the United States.^3^, ^17^, ^18^ High use of flaxseed, omega‐3 fatty acids, and megavitamin therapy was also reported in patients with lysosomal storage diseases.^11^ High use of these therapies may be due to their accessibility, affordability, higher prevalence in the media, and recommendations from family and friends. Megavitamin therapy is also known to be recommended by physicians for mitochondrial disorders. The relatively low spending on CAM ($100 CAD or less per month) may be attributable to the universal healthcare system and private health insurance options that cover CAM therapies in Canada.

Participants who used CAM were found to have no significant differences in age, sex, or education level. Previous studies have demonstrated increased CAM use in females, adults, and those with higher education when compared to CAM Non‐users.^11^, ^21^, ^22^ Comparisons between CAM Users and CAM Non‐users were not performed due to our limited sample size, therefore making the study underpowered to adequately analyze these comparisons. These comparisons are warranted in future studies.

Overall, the CAM Users of this study viewed CAM favorably and found their CAM therapies effective. Due to the various manifestations and symptoms that may present in different IEM disorders, it is difficult to generalize the effectiveness of CAM therapies for all patients with IEM. Additionally, with the rise of integrative health and evidence‐based medicine, certain CAM therapies may not be considered CAM for certain IEMs. More controlled studies should be performed to further understand the effectiveness of CAM therapies in patients with IEM.

A total of 40% of participants had not discussed their CAM use with a conventional HCP. This finding is higher than in the study by Balwani et al, but lower than Erdol and Saglam; these differences may be due to variations in acceptance and awareness towards CAM in different cultures. Of those who had discussed their CAM use, one‐third had not discussed it with their physician. Additionally, no adults had discussed their CAM use with a dietician, a HCP who plays a significant role in the care of many patients with IEM. It is likely that some patients decide to use CAM based on the Internet or personal anecdotes. A lack of consistent communication between patients and HCPs could lead to preventable adverse events and drug interactions. For example, harm from chiropractic manipulation has been reported in children with underlying medical pathology^25^; patients with IEM who have osteopenia or osteoporosis are at particular risk. Large doses of omega‐3 fatty acids may decrease platelet aggregation and, thus, increase bleeding time; this may be harmful in glycogen storage diseases that are associated with platelet function defects.^26^ Some herbal therapies have also been associated with various renal syndromes.^13^ Nonetheless, all HCPs are encouraged to provide an open and judgment‐free environment to discuss CAM in order to provide safe and patient‐centered care to patients with IEM.

### 4.1 Limitations

Our sample size was smaller than other similar studies, thereby impacting our study's external validity and power. Selection bias was possible as we had a distribution of IEM disorders that may not be representative of this patient population. There may also be recall bias due to the nature of the questionnaire questions. Lastly, as an exploratory study, we were unable to provide conclusive evidence on the effectiveness of CAM therapies for patients with IEM. However, our findings should encourage larger, controlled trials on the safety and efficacy of CAM in patients with IEM to be performed.

## 5 CONCLUSION

CAM is commonly used in patients with IEM. Overall, patients with IEM and caregivers view their CAM therapies as effective; however, many patients are not informing their physicians and allied health care team about their CAM use. Therefore, HCPs are encouraged to stay informed about CAM, openly discuss CAM use with their patients in order to evaluate safety, and advocate for the development of further research in this area.

## CONFLICT OF INTEREST

All authors declare that they have no conflict of interest regarding the contents of this manuscript.

## AUTHOR CONTRIBUTIONS

J.T. was involved in the study conception and design, data collection and analysis, and writing of this manuscript. C.A.R. was involved in the study design and revision of this manuscript. M.R.M. was involved in the study design, data analysis, and revision of this manuscript. S.R. was involved in the study design and revision of this manuscript. C.P. (Guarantor) was involved in the study conception and design, data collection and analysis, and revision of this manuscript.

## COMPLIANCE WITH ETHICS GUIDELINES: A CONCISE 1 SENTENCE TAKE‐HOME MESSAGE (SYNOPSIS) OF THE ARTICLE, OUTLINING WHAT THE READER LEARNS FROM THE ARTICLE

This article describes the common use and perceived effectiveness of complementary and alternative medicine in patients with inborn errors of metabolism from a single metabolic center in Canada, and the factors associated with using these therapies.

## DETAILS OF ETHICS APPROVAL

This study was approved by the Western University Research Ethics Board and Lawson Health Research Institute (London, Ontario, Canada). IRB 00000940, HSREB File Number: 109460.

## PATIENT CONSENT STATEMENT

All procedures followed were in accordance with the ethical standards of the responsible committee on human experimentation (institutional and national) and with the Helsinki Declaration of 1975, as revised in 2000 (5). Informed consent was obtained from all patients for being included in the study. No identifying information was collected in the study.

## Supporting information


**Appendix S1**: Supporting information

## Data Availability

All paper files are stored in the London Health Sciences Centre (LHSC) Medical Genetics Department. Data without identifiers are stored in a secure LHSC hospital network drive. Only the authors have access to these files.
