# Supplementary material for: Use of complementary and alternative medicine in patients with inborn errors of metabolism: A single‐center study
Source: JIMD Rep. 2019 Dec 18;51(1):105–12. doi: 10.1002/jmd2.12089 (PMC7012736; doi:10.1002/jmd2.12089)
Supplement: Supplementary file 1 — Appendix S1: Supporting information [file JMD2-51-105-s001.docx]

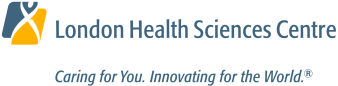


**USE OF COMPLEMENTARY AND ALTERNATIVE MEDICINE IN PATIENTS WITH INBORN ERRORS OF METABOLISM**

**QUESTIONNAIRE**

**Please note the following:**

- Please complete this questionnaire after you have read and understood the Letter of Information and Consent included. Make sure all of your questions have been answered to your satisfaction before completing this questionnaire.
- Please complete the information requested as completely as possible. All questions are in relation to the patient diagnosed with an inborn error of metabolism (unless otherwise specified).
- If the patient is under the age of 13 years or is incapable of completing this questionnaire, please have a parent/legal guardian complete the questionnaire on the patient’s behalf.
- Information contained in this questionnaire is confidential and will be stored in a secure research database (housed by the London Health Sciences Centre).
- Please ask the research team if you are having difficulty completing this questionnaire or have questions about the information being gathered for this study.

Thank you very much for your time in participating in our research study.

**Today’s date (YYYY/MM/DD): _____________ / ________ / ________**

1. **DEMOGRAPHIC INFORMATION**

| **Age of Patient:** | 0–17 years old  18–70 years old | | | | **Gender:** | Male  Female  Other |
| --- | --- | --- | --- | --- | --- | --- |
| **Ethnicity:** | | Caucasian  Black  Latin American  South Asian  Chinese | | South East Asian  Arab  Aboriginal  Other: ______________________________________ | | |
| **Highest level of  education completed:** | | | No schooling completed  Grade school (K-Grade 8)  Some high school, no diploma  High school diploma  Some university/college, no degree  Trade/technical/vocational training  Associate degree  Bachelor’s degree  Graduate/professional degree  Other: _____________________________________________________ | | | |

**If a parent/legal guardian is completing this questionnaire on the patient’s behalf, please provide the following information (please leave blank otherwise):**

| **Relationship to Patient:** | ___________________________________________________________________ |
| --- | --- |
| **Highest level of education completed:** | No schooling completed  Grade school (K-Grade 8)  Some high school, no diploma  High school diploma  Some university/college, no degree  Trade/technical/vocational training  Associate degree  Bachelor’s degree  Graduate/professional degree  Other: __________________________________________________________ |

1. **MEDICAL INFORMATION**

**Reminder:**

- *In this questionnaire, the word “you” always refers to the study participant (i.e. the patient diagnosed with an inborn error of metabolism).*

| **What is your specific diagnosis?:** | |  | | | | | |
| --- | --- | --- | --- | --- | --- | --- | --- |
| **Age when**  **diagnosed:** |  | | | |  | | |
| **Current medical therapy prescribed (please list all current medications):** | | | | | | | |
| **Are you on Enzyme Replacement Therapy (ERT)?:** | | | | Yes  No | | **Have you had a Bone Marrow Transplant?:** | Yes  No |
| **Other medical therapies**  **(please specify):** | | |  | | | | |

1. **USE OF COMPLEMENTARY & ALTERNATIVE MEDICINE (CAM)**

**Please note:**

- “*CAM*” stands for “*Complementary and Alternative Medicine*”.
- “*IEM”* stands for *“Inborn Errors of Metabolism”.*

| - 1. **Have you ever used a CAM therapy to help your IEM diagnosis?:**   CAMs are a diverse range of health-related therapies and products that are currently not considered to be part of conventional medicine. These include, but are not limited to:   \| *Acupressure*  *Acupuncture*  *Aromatherapy/essential oils*  *Ayurveda*  *Biofeedback*  *Chelation therapy*  *Chiropractic*  *Commercial diet*  *Cupping therapy*  *Energy healing/Reiki*  *Folk medicine* \| *Guided imagery*  *Herbal medicine*  *Homeopathy*  *Hypnosis*  *Lifestyle diet*  *Magnetic therapy*  *Massage therapy*  *Meditation*  *Megavitamin therapy*  *Naturopathy*  *Nonvitamin, nonmineral, natural products* \| *Osteopathy*  *Reflexology*  *Relaxation techniques*  *Self-help group*  *Spiritual healing*  *Tai chi*  *Traditional Chinese Medicine*  *Water therapy*  *Yoga* \| \| --- \| --- \| --- \| | |
| --- | --- | --- | --- | --- |
| **Yes  No  Would rather not specify** | **e** |

If indicated **“*YES”*** to **3.1)**, please answer questions **3.2), 3.3), 3.4) and 3.5)** (please leave blank otherwise):

| - 1. **What are your reasons for using CAMs? (please indicate all that apply):** | To relieve symptoms  To complement my prescribed medical therapy  Not satisfied with my prescribed medical therapy  To combat the prescribed medical therapy’s side effect(s)  Prescribed medical therapy is too expensive  Suggested by a conventional medical professional  To try something new/different  My CAM therapies make me feel better  Other: ____________________________________________ |
| --- | --- |
|  |  |

| - 1. **How did you learn about CAMs? (please indicate all that apply):** | Health care professional  Family member/friend  Internet/social media  TV  Radio  Newspaper/magazine  Other:_____________________________________________ |
| --- | --- |
|  |  |

| - 1. **On average, how much do you spend on CAMs per month?** | | | $0–$100  $100–$200  $200–$400  $400–$600  $600–$800  $800–$1000  $1000+ | | |  |
| --- | --- | --- | --- | --- | --- | --- |
|  | | |  |  |  |  |
| - 1. **Have you discussed your CAM use with a health care professional?:**   Yes  No  Would rather not specify | | | | | | |
|  | | **3.5.1) If indicated “*YES”* to 3.5), which health care professional(s) have you discussed your CAM use with? (please indicate all that apply):** | | | | |
|  | | Physician  Nurse Practitioner | Registered Nurse  Pharmacist | | Physiotherapist  Dietician | Genetic Counsellor  Other: _______________________ |
|  | |  |  | | | |
|  | | **3.5.2) If indicated “*NO”* to 3.5), why have you not discussed your CAM use with a health care professional? (please indicate all that apply):**  Uncomfortable discussing my CAM use  Health care professional never asked about it  Afraid health care professional will disapprove  Health care professional lacks knowledge about the CAM therapy  Other: ______________________________________________________________________________ | | | | |

1. **SUPPLEMENTS**

Please indicate if you have taken each supplement in the past to help your IEM diagnosis, and if so, how effective you found the supplement. If you have not taken one of the listed supplements before or are unsure, please leave its corresponding *effectiveness* question blank.

| - 1. **ANTIOXIDANTS**   **Taken in the past:**  Yes  No  Unsure | | |  | | | |
| --- | --- | --- | --- | --- | --- | --- |
| If indicated **“*YES”***, on a scale of **0 to 5**, how **effective** was this supplement for you? (please circle): | | | | | | |
| Not effective  at all |  |  | |  |  | Very  effective |
| **0** | **1** | **2** | | **3** | **4** | **5** |

| - 1. **ECHINACEA**   **Taken in the past:**  Yes  No  Unsure | | |  | | | |
| --- | --- | --- | --- | --- | --- | --- |
| If indicated **“*YES”***, on a scale of **0 to 5**, how **effective** was this supplement for you? (please circle): | | | | | | |
| Not effective  at all |  |  | |  |  | Very  effective |
| **0** | **1** | **2** | | **3** | **4** | **5** |

| - 1. **FENUGREEK**   **Taken in the past:**  Yes  No  Unsure | | |  | | | |
| --- | --- | --- | --- | --- | --- | --- |
| If indicated **“*YES”***, on a scale of **0 to 5**, how **effective** was this supplement for you? (please circle): | | | | | | |
| Not effective  at all |  |  | |  |  | Very  effective |
| **0** | **1** | **2** | | **3** | **4** | **5** |

| - 1. **FLAXSEED**   **Taken in the past:**  Yes  No  Unsure | | |  | | | |
| --- | --- | --- | --- | --- | --- | --- |
| If indicated **“*YES”***, on a scale of **0 to 5**, how **effective** was this supplement for you? (please circle): | | | | | | |
| Not effective  at all |  |  | |  |  | Very  effective |
| **0** | **1** | **2** | | **3** | **4** | **5** |

| - 1. **GARLIC SUPPLEMENTS**   **Taken in the past:**  Yes  No  Unsure | | |  | | | |
| --- | --- | --- | --- | --- | --- | --- |
| If indicated **“*YES”***, on a scale of **0 to 5**, how **effective** was this supplement for you? (please circle): | | | | | | |
| Not effective  at all |  |  | |  |  | Very  effective |
| **0** | **1** | **2** | | **3** | **4** | **5** |

| - 1. **GENISTEIN**   **Taken in the past:**  Yes  No  Unsure | | |  | | | |
| --- | --- | --- | --- | --- | --- | --- |
| If indicated **“*YES”***, on a scale of **0 to 5**, how **effective** was this supplement for you? (please circle): | | | | | | |
| Not effective  at all |  |  | |  |  | Very  effective |
| **0** | **1** | **2** | | **3** | **4** | **5** |

| - 1. **GINKGO**   **Taken in the past:**  Yes  No  Unsure | | |  | | | |
| --- | --- | --- | --- | --- | --- | --- |
| If indicated **“*YES”***, on a scale of **0 to 5**, how **effective** was this supplement for you? (please circle): | | | | | | |
| Not effective  at all |  |  | |  |  | Very  effective |
| **0** | **1** | **2** | | **3** | **4** | **5** |

| - 1. **GINSENG**   **Taken in the past:**  Yes  No  Unsure | | |  | | | |
| --- | --- | --- | --- | --- | --- | --- |
| If indicated **“*YES”***, on a scale of **0 to 5**, how **effective** was this supplement for you? (please circle): | | | | | | |
| Not effective  at all |  |  | |  |  | Very  effective |
| **0** | **1** | **2** | | **3** | **4** | **5** |

| - 1. **GLUCOSAMINE (only)**   **Taken in the past:**  Yes  No  Unsure | | |  | | | |
| --- | --- | --- | --- | --- | --- | --- |
| If indicated **“*YES”***, on a scale of **0 to 5**, how **effective** was this supplement for you? (please circle): | | | | | | |
| Not effective  at all |  |  | |  |  | Very  effective |
| **0** | **1** | **2** | | **3** | **4** | **5** |

| - 1. **GLUCOSAMINE + CHONDROITIN**   **Taken in the past:**  Yes  No  Unsure | | |  | | | |
| --- | --- | --- | --- | --- | --- | --- |
| If indicated **“*YES”***, on a scale of **0 to 5**, how **effective** was this supplement for you? (please circle): | | | | | | |
| Not effective  at all |  |  | |  |  | Very  effective |
| **0** | **1** | **2** | | **3** | **4** | **5** |

| - 1. **MEGAVITAMIN THERAPY**   **Taken in the past:**  Yes  No  Unsure | | |  | | | |
| --- | --- | --- | --- | --- | --- | --- |
| If indicated **“*YES”***, on a scale of **0 to 5**, how **effective** was this supplement for you? (please circle): | | | | | | |
| Not effective  at all |  |  | |  |  | Very  effective |
| **0** | **1** | **2** | | **3** | **4** | **5** |

| - 1. **METHYLSULFONYLMETHANE (MSM)**   **Taken in the past:**  Yes  No  Unsure | | |  | | | |
| --- | --- | --- | --- | --- | --- | --- |
| If indicated **“*YES”***, on a scale of **0 to 5**, how **effective** was this supplement for you? (please circle): | | | | | | |
| Not effective  at all |  |  | |  |  | Very  effective |
| **0** | **1** | **2** | | **3** | **4** | **5** |

| - 1. **OMEGA-3 FATTY ACIDS**   **Taken in the past:**  Yes  No  Unsure | | |  | | | |
| --- | --- | --- | --- | --- | --- | --- |
| If indicated **“*YES”***, on a scale of **0 to 5**, how **effective** was this supplement for you? (please circle): | | | | | | |
| Not effective  at all |  |  | |  |  | Very  effective |
| **0** | **1** | **2** | | **3** | **4** | **5** |

| - 1. **PREBIOTICS**   **Taken in the past:**  Yes  No  Unsure | | |  | | | |
| --- | --- | --- | --- | --- | --- | --- |
| If indicated **“*YES”***, on a scale of **0 to 5**, how **effective** was this supplement for you? (please circle): | | | | | | |
| Not effective  at all |  |  | |  |  | Very  effective |
| **0** | **1** | **2** | | **3** | **4** | **5** |

| - 1. **PROBIOTICS**   **Taken in the past:**  Yes  No  Unsure | | |  | | | |
| --- | --- | --- | --- | --- | --- | --- |
| If indicated **“*YES”***, on a scale of **0 to 5**, how **effective** was this supplement for you? (please circle): | | | | | | |
| Not effective  at all |  |  | |  |  | Very  effective |
| **0** | **1** | **2** | | **3** | **4** | **5** |

| - 1. **OTHER** | | |  | | | |
| --- | --- | --- | --- | --- | --- | --- |
| **Please specify: ____________________________________________________________________________** | | | | | | |
| On a scale of **0 to 5**, how **effective** was this supplement for you? (please circle): | | | | | | |
| Not effective  at all |  |  | |  |  | Very  effective |
| **0** | **1** | **2** | | **3** | **4** | **5** |

1. **TREATMENTS**

Please indicate if you have used each treatment in the past to help your IEM diagnosis, and if so, how effective you found the treatment. If you have not used one of the listed treatments before or are unsure, please leave its corresponding *effectiveness* question blank.

| - 1. **ACUPRESSURE**   **Used in the past:**  Yes  No  Unsure | | |  | | | |
| --- | --- | --- | --- | --- | --- | --- |
| If indicated **“*YES”***, on a scale of **0 to 5**, how **effective** was this treatment for you? (please circle): | | | | | | |
| Not effective  at all |  |  | |  |  | Very  effective |
| **0** | **1** | **2** | | **3** | **4** | **5** |

| - 1. **ACUPUNCTURE**   **Used in the past:**  Yes  No  Unsure | | |  | | | |
| --- | --- | --- | --- | --- | --- | --- |
| If indicated **“*YES”***, on a scale of **0 to 5**, how **effective** was this treatment for you? (please circle): | | | | | | |
| Not effective  at all |  |  | |  |  | Very  effective |
| **0** | **1** | **2** | | **3** | **4** | **5** |
| - 1. **AROMATHERAPY/ESSENTIAL OILS**   **Used in the past:**  Yes  No  Unsure | | |  | | | |
| If indicated **“*YES”***, on a scale of **0 to 5**, how **effective** was this treatment for you? (please circle): | | | | | | |
| Not effective  at all |  |  | |  |  | Very  effective |
| **0** | **1** | **2** | | **3** | **4** | **5** |

| - 1. **AYURVEDA**   **Used in the past:**  Yes  No  Unsure | | |  | | | |
| --- | --- | --- | --- | --- | --- | --- |
| If indicated **“*YES”***, on a scale of **0 to 5**, how **effective** was this treatment for you? (please circle): | | | | | | |
| Not effective  at all |  |  | |  |  | Very  effective |
| **0** | **1** | **2** | | **3** | **4** | **5** |

| - 1. **BIOFEEDBACK**   **Used in the past:**  Yes  No  Unsure | | |  | | | |
| --- | --- | --- | --- | --- | --- | --- |
| If indicated “***YES”***, on a scale of **0 to 5**, how **effective** was this treatment for you? (please circle): | | | | | | |
| Not effective  at all |  |  | |  |  | Very  effective |
| **0** | **1** | **2** | | **3** | **4** | **5** |

| - 1. **CHIROPRACTIC**   **Used in the past:**  Yes  No  Unsure | | |  | | | |
| --- | --- | --- | --- | --- | --- | --- |
| If indicated **“*YES”***, on a scale of **0 to 5**, how **effective** was this treatment for you? (please circle): | | | | | | |
| Not effective  at all |  |  | |  |  | Very  effective |
| **0** | **1** | **2** | | **3** | **4** | **5** |

| - 1. **CUPPING THERAPY**   **Used in the past:**  Yes  No  Unsure | | |  | | | |
| --- | --- | --- | --- | --- | --- | --- |
| If indicated **“*YES”***, on a scale of **0 to 5**, how **effective** was this treatment for you? (please circle): | | | | | | |
| Not effective  at all |  |  | |  |  | Very  effective |
| **0** | **1** | **2** | | **3** | **4** | **5** |

| - 1. **ENERGY HEALING/REIKI**   **Used in the past:**  Yes  No  Unsure | | |  | | | |
| --- | --- | --- | --- | --- | --- | --- |
| If indicated **“*YES”***, on a scale of **0 to 5**, how **effective** was this treatment for you? (please circle): | | | | | | |
| Not effective  at all |  |  | |  |  | Very  effective |
| **0** | **1** | **2** | | **3** | **4** | **5** |

| - 1. **GUIDED IMAGERY**   **Used in the past:**  Yes  No  Unsure | | |  | | | |
| --- | --- | --- | --- | --- | --- | --- |
| If indicated **“*YES”***, on a scale of **0 to 5**, how **effective** was this treatment for you? (please circle): | | | | | | |
| Not effective  at all |  |  | |  |  | Very  effective |
| **0** | **1** | **2** | | **3** | **4** | **5** |

| - 1. **HOMEOPATHY**   **Used in the past:**  Yes  No  Unsure | | |  | | | |
| --- | --- | --- | --- | --- | --- | --- |
| If indicated **“*YES”***, on a scale of **0 to 5**, how **effective** was this treatment for you? (please circle): | | | | | | |
| Not effective  at all |  |  | |  |  | Very  effective |
| **0** | **1** | **2** | | **3** | **4** | **5** |

| - 1. **MAGNETIC THERAPY**   **Used in the past:**  Yes  No  Unsure | | |  | | | |
| --- | --- | --- | --- | --- | --- | --- |
| If indicated **“*YES”***, on a scale of **0 to 5**, how **effective** was this treatment for you? (please circle): | | | | | | |
| Not effective  at all |  |  | |  |  | Very  effective |
| **0** | **1** | **2** | | **3** | **4** | **5** |

| - 1. **MASSAGE THERAPY**   **Used in the past:**  Yes  No  Unsure | | |  | | | |
| --- | --- | --- | --- | --- | --- | --- |
| If indicated **“*YES”***, on a scale of **0 to 5**, how **effective** was this treatment for you? (please circle): | | | | | | |
| Not effective  at all |  |  | |  |  | Very  effective |
| **0** | **1** | **2** | | **3** | **4** | **5** |

| - 1. **MEDITATION**   **Used in the past:**  Yes  No  Unsure | | |  | | | |
| --- | --- | --- | --- | --- | --- | --- |
| If indicated **“*YES”***, on a scale of **0 to 5**, how **effective** was this treatment for you? (please circle): | | | | | | |
| Not effective  at all |  |  | |  |  | Very  effective |
| **0** | **1** | **2** | | **3** | **4** | **5** |

| - 1. **NATUROPATHY**   **Used in the past:**  Yes  No  Unsure | | |  | | | |
| --- | --- | --- | --- | --- | --- | --- |
| If indicated **“*YES”***, on a scale of **0 to 5**, how **effective** was this treatment for you? (please circle): | | | | | | |
| Not effective  at all |  |  | |  |  | Very  effective |
| **0** | **1** | **2** | | **3** | **4** | **5** |

| - 1. **OSTEOPATHY**   **Used in the past:**  Yes  No  Unsure | | |  | | | |
| --- | --- | --- | --- | --- | --- | --- |
| If indicated **“*YES”***, on a scale of **0 to 5**, how **effective** was this treatment for you? (please circle): | | | | | | |
| Not effective  at all |  |  | |  |  | Very  effective |
| **0** | **1** | **2** | | **3** | **4** | **5** |

| - 1. **PROGRESSIVE RELAXATION**   **Used in the past:**  Yes  No  Unsure | | |  | | | |
| --- | --- | --- | --- | --- | --- | --- |
| If indicated **“*YES”***, on a scale of **0 to 5**, how **effective** was this treatment for you? (please circle): | | | | | | |
| Not effective  at all |  |  | |  |  | Very  effective |
| **0** | **1** | **2** | | **3** | **4** | **5** |

| - 1. **REFLEXOLOGY**   **Used in the past:**  Yes  No  Unsure | | |  | | | |
| --- | --- | --- | --- | --- | --- | --- |
| If indicated **“*YES”***, on a scale of **0 to 5**, how **effective** was this treatment for you? (please circle): | | | | | | |
| Not effective  at all |  |  | |  |  | Very  effective |
| **0** | **1** | **2** | | **3** | **4** | **5** |

| - 1. **SPIRITUAL HEALING BY OTHERS**   **Used in the past:**  Yes  No  Unsure | | |  | | | |
| --- | --- | --- | --- | --- | --- | --- |
| If indicated **“*YES”***, on a scale of **0 to 5**, how **effective** was this treatment for you? (please circle): | | | | | | |
| Not effective  at all |  |  | |  |  | Very  effective |
| **0** | **1** | **2** | | **3** | **4** | **5** |

| - 1. **TRADITIONAL CHINESE MEDICINE**   **Used in the past:**  Yes  No  Unsure | | |  | | | |
| --- | --- | --- | --- | --- | --- | --- |
| If indicated **“*YES”***, on a scale of **0 to 5**, how **effective** was this treatment for you? (please circle): | | | | | | |
| Not effective  at all |  |  | |  |  | Very  effective |
| **0** | **1** | **2** | | **3** | **4** | **5** |

| - 1. **WATER THERAPY**   **Used in the past:**  Yes  No  Unsure | | |  | | | |
| --- | --- | --- | --- | --- | --- | --- |
| If indicated **“*YES”***, on a scale of **0 to 5**, how **effective** was this treatment for you? (please circle): | | | | | | |
| Not effective  at all |  |  | |  |  | Very  effective |
| **0** | **1** | **2** | | **3** | **4** | **5** |

| - 1. **YOGA**   **Used in the past:**  Yes  No  Unsure | | |  | | | |
| --- | --- | --- | --- | --- | --- | --- |
| If indicated **“*YES”***, on a scale of **0 to 5**, how **effective** was this treatment for you? (please circle): | | | | | | |
| Not effective  at all |  |  | |  |  | Very  effective |
| **0** | **1** | **2** | | **3** | **4** | **5** |

| - 1. **OTHER** | | |  | | | |
| --- | --- | --- | --- | --- | --- | --- |
| **Please specify: ____________________________________________________________________________** | | | | | | |
| On a scale of **0 to 5**, how **effective** was this treatment for you? (please circle): | | | | | | |
| Not effective  at all |  |  | |  |  | Very  effective |
| **0** | **1** | **2** | | **3** | **4** | **5** |

Thank you very much for completing the questionnaire. **Please return the questionnaire back to the research team.**

| **For research staff use only** | |
| --- | --- |
| **Study ID:** | |
| **Time to complete questionnaire (minutes):** | |
| **Intake completed:** | |
| **By:** | **Date:** |
